# Consecutive Reaction to Construct Hierarchical Nanocrystalline CuS “Branch” with Tunable Catalysis Properties

**DOI:** 10.1038/srep30604

**Published:** 2016-07-28

**Authors:** Xiangdan Zhang, Feifei Yang, Shizhong Cui, Wutao Wei, Weihua Chen, Liwei Mi

**Affiliations:** 1 Center for Advanced Materials Research, Zhongyuan University of Technology, Zhengzhou, Henan 450007, P.R. China; 2 College of Chemistry and Molecular Engineering, Zhengzhou University, Zhengzhou, Henan 450001, P.R. China

## Abstract

New CuS nanocrystals with a 3D hierarchical branched structure are successfully synthesized through *in situ* consecutive reaction method with copper foam as template. The formation mechanism of the 3D hierarchical branched structure obtained from the secondary reaction is investigated by adjusting the reaction time. The morphology of CuS nanosheet arrays with the 3D hierarchical branched structure is changed through Cu^2+^ exchange. In this method, the copper foam reacted completely, and the as-synthesized CuS@Cu_9_S_5_ nanocrystals are firmly grown on the surface of the 3D framework. This tunable morphology significantly influence the physical and chemical properties, particularly catalytic performance, of the materials. The as-obtained material of Cu@CuS-2 with the 3D hierarchical branched structure as catalyst for methylene blue degradation exhibits good catalytic performance than that of the material of Cu@CuS with 2D nanosheets in dark environment. Furthermore, the cation exchange between Cu and Cu^2+^ indicates that Cu^2+^ in wastewater could be absorbed by Cu@CuS-2 with the 3D hierarchical branched structure. The exchanged resultant of CuS@Cu_9_S_5_ retains its capability to degrade organic dyes. This *in situ* consecutive reaction method may have a significant impact on controlling the crystal growth direction of inorganic material.

Micro/nano materials with a 3D hierarchical structure are fabricated using nanowires^1^,^2^,^3^ and nanosheets^4^,^5^,^6^ as building blocks, these materials have gained increased interest because of their promising structure with large surface area and sufficient surface active sites^4^,^7^,^8^. Generally, the architecture of inorganic materials is related to their properties. Compared with individual nanowires or nanosheets with 1D or 2D structure, materials with 3D hierarchical micro/nanostructures can provide more ion transfer paths and stable structure, which are beneficial to promote application properties. Therefore, 3D hierarchical structures have received increased research attention because of increasing demand for high-performance materials in recent years.

Currently, numerous studies mainly focused on preparation of inorganic materials with 3D hierarchical structures^9^,^10^,^11^,^12^,^13^,^14^,^15^. Nevertheless, designing an easy and controllable method for prepare these materials remains a challenge. The crystal growth process of materials can be terminated by controlling the reaction condition while maintaining the partial reaction of the reactant. The secondary reaction can be introduced by manipulating the nucleation and growth processes by using the aforementioned product with unreacted raw materials as precursor. The secondary reaction may change the preferential crystal growth direction, thereby facilitating the fabrication of a 3D hierarchical structure.

As one of the most important p-type semiconductors, CuS has attracted significant research attention because of their potential applications in photocatalysis^16^,^17^,^18^, solar cell devices^19^,^20^,^21^, biosensors^22^,^23^,^24^ and supercapacitor^25^,^26^. Moreover, several methods have been investigated to synthesize CuS with 3D hierarchical structure, such as oil-water interface method^27^, oriented attachment^18^, sacrificial templating^28^,^29^, solvothermal method^30^. However, these one-step synthesis methods are uncontrollable, and few report pay close attention to synthesizing 3D hierarchical structure by *in situ* consecutive reaction. For instance, Zhao and co-worker^31^ have prepared flower-like CuS hollow microspheres via solvothermal method with CuSO_4_•5H_2_O as reactant, but this cuprate can’t be used to conduct the secondary reaction for the amorphous structure. In our previous work^32^, CuS microflowers with 3D hierarchal structure have been synthesized using copper foam as template by one-step solvothermal method, while the formation of microflowers is uncontrollable. Therefore, copper foam with a stable 3D framework can be used as a promising candidate to conduct *in situ* consecutive reaction. In the first reaction, a layer of CuS nanosheets with a 2D structure are grown on the surface of unreacted copper foam by controlling reaction time and temperature. Cu@CuS can be used as a precursor and continuously reacted using the method that similar to the first reaction. Subsequently, the crystal growth direction maybe changed during the secondary reaction. Consequently, the newly formed nanosheets are assembled with nanosheets that obtained from the first reaction to construct the 3D hierarchical structure.

Cation exchange is a promising technique used to generate inorganic materials with distinct structures and manipulate the composition of as-synthesized nanocrystals^33^. The exchange process includes the replacement of the cations in the precursor, such as metal selenides or sulfides (CdSe or CdS) by other cations (e.g., Ag^+^, Cd^+^, Cu^+^, Pb^+^) in the specific solvents^34^,^35^. Many studies synthesized a novel heterostructure through cation exchange. For example, Han *et al*.^36^ synthesized a CuS mesostructure through cation exchange of Cd^2+^ and maintained its original morphology. Donega *et al*.^37^ synthesized CulnS_2_ nanocrystals through partial indium-cation exchange into small Cu_2_−_X_S dot-like nanocrystals. However, cation exchange between the similar elements with differ valent state has been rarely reported. Thus, we intend to synthesize a new material through cation exchange between Cu and Cu^2+^ to optimize the initial material with 3D hierarchical structure and change their original morphology.

In this paper, we report a *in situ* consecutive reaction method with copper foam as template to effectively control the crystal growth direction of CuS. First, Cu@CuS nanosheets with 2D curly structure were synthesized through a traditional solvothermal method at 100 °C for 24 h. Subsequently, the as-obtained dry material was used as precursor to continue the reaction. Consequently, Cu@CuS nanosheet arrays with 3D hierarchical branched structure were synthesized. Furthermore, the third reaction was conducted by using the resultant of the secondary reaction as precursor to prepare material with the similar 3D hierarchical branched structure, which called morphological heredity. Meanwhile, the as-synthesized resultant of the secondary reaction was also used as a template to fabricate a series of products with different morphologies through Cu^2+^ exchange. This *in situ* consecutive reaction method has never been reported, such method presents the following advantages: (1) provides a facile, controllable method to synthesize 3D hierarchical structure, (2) creates an excellent morphology for each reaction, (3) features tunable crystal structures to maximize the properties of the each material and achieve their complementary advantages, (4) copper template participated in each reaction and reacts completely in the final reaction, thereby decreasing the amount of raw materials used. This tunable 3D hierarchical branched structure provides a large of specific surface area and more dispersed active sites when used as catalyst.

## Experimental Section

### Materials

All the chemicals and solvents in this work were of analytic grade purity and used without further purification.

### Synthesis of Cu@CuS Nanosheets with 2D Curly Structure

The Cu@CuS nanosheets with 2D curly structure were successfully fabricated via a facial solvothermal reaction using copper foam as template. Typically, about 3 mmol sulfur powder was added into 16 ml N, N-Dimethylformamide (DMF) in a 30 ml Teflon-lined autoclave. The solution was stirred for a few minutes at room temperature until the sulfur powder was dispersed uniformly in the DMF. Subsequently, a piece of copper foam (1 cm × 1 cm, 1.5 mmol) was placed in the solution, and the autoclave kept at 100 °C for 24 h. After reaction, the solution was cooled down to room temperature naturally. The dark Cu@CuS product was washed thoroughly with deionized water and 95% ethanol for several times, and dried in a vacuum oven at 60 °C for 8 h.

### *In Situ* Consecutive Reaction for Controllable Synthesis Cu@CuS Nanosheet Arrays with 3D Hierarchical Branched Structure

The Cu@CuS nanosheet arrays with 3D hierarchical branched structure were fabricated using as-synthesized Cu@CuS as a precursor. In this process, a time-dependent experiment was performed using the method that similar to the first reaction. The autoclave was kept at 100 °C for 0.5, 1, 2, 4, 6, 8, 10, 12 h to synthesize Cu@CuS-0.5, Cu@CuS-1, Cu@CuS-2, Cu@CuS-4, Cu@CuS-6, Cu@CuS-8, Cu@CuS-10, Cu@CuS-12, respectively. The amount of sulfur powder used was half of the amount used in the first reaction.

The effect of the amount of sulfur powder in the secondary reaction was also investigated to obtain an optimized morphology. The amount of sulfur powder was adjusted to 1 and 3 mmol, and the other conditions were constant, the temperature was maintained at 100 °C for 2 h. The products obtained were Cu@CuS-2-1 and Cu@CuS-2-2.

The third reaction was conducted using Cu@CuS-2 as a precursor to further investigate the crystal growth direc-tion of CuS nanosheet arrays with 3D hierarchical branched structure. Approximately 0.15 mmol sulfur powder solution was dispersed in 16 ml of DMF at 100 °C for 2 h to obtain Cu@CuS-2-3.

### Cation Exchange for Synthesis of CuS@Cu_9_S_5_

The products of CuS@Cu_9_S_5_ with multiple morphologies were fabricated using the as-synthesized Cu@CuS-2 as a precursor in different solvent systems. Typically, about 3 mmol copper nitrate trihydrate solution was dissolved in 10 ml of DMF and 6 ml of ethylene glycol in a 30 ml Teflon-lined autoclave. The solution was stirred for a few minutes at room temperature until Cu^2+^ was dissolved completely. Subsequently, the Cu@CuS-2 was placed in the solution and autoclaved at 160 °C for 16 h to obtain CuS@Cu_9_S_5_-1. We also prepared another sample of CuS@Cu_9_S_5_-2 by using 16 ml of DMF as solvent with the same reaction conditions of CuS@Cu_9_S_5_-1. Finally, all the products were washed several times with deionized water and 95% ethanol, dried in a vacuum oven at 60 °C for 8 h, and collected for further characterization.

### Characterization

X-ray diffraction (XRD) patterns were recorded on Bruker D8 Advance X-ray powder diffractometer with Cu-Kα irradiation at a scan rate of 0.1° s^−1^. All XRD measurements were performed within 20° ≤ 2θ ≤ 80°. Morphologies and sizes of the as-synthesized products were characterized using Zeiss Merlin Compact scanning electron microscope (SEM) equipped with an energy dispersive X-ray spectroscopy (EDX) system. The nanostructures of the resulting materials were also recorded with a JEOL JEM-2010 transmission electron microscope (TEM). The X-ray photoelectron spectroscopy (XPS) of the as-synthesized CuS crystals was collected using a Kratos AXIS ULTRA X-ray photoelectron microscope with Al-Kα X-rays as the excitation source.

### Catalytic activity evaluation

MB and RB are effective chemicals used to evaluate the catalytic performance. Typically, a piece of dry sample used as catalyst was combined with MB or RB dye solution (10 mg L^−1^, 30 mL). The reaction was initiated by adding H_2_O_2_ (10 mL) and proceeded at 30 °C with slow agitation in a dark environment. The concentration of MB or RB was determined with a UV-vis spectrophotometer (PerkinElmer Lambda 35). At each sampling time (about 15 min), about 3 mL of solution was removed from the MB or RB solution and immediately used for analysis. After the reaction, the catalyst was removed from the solution using a tweezer and washed with distilled water and 95% ethanol for further characterization. The mixed solution of MB@RB was degraded by the Cu@CuS or CuS@Cu_9_S_5_ catalysts in the same manner.

## Results and Discussion

**Figure 1:**
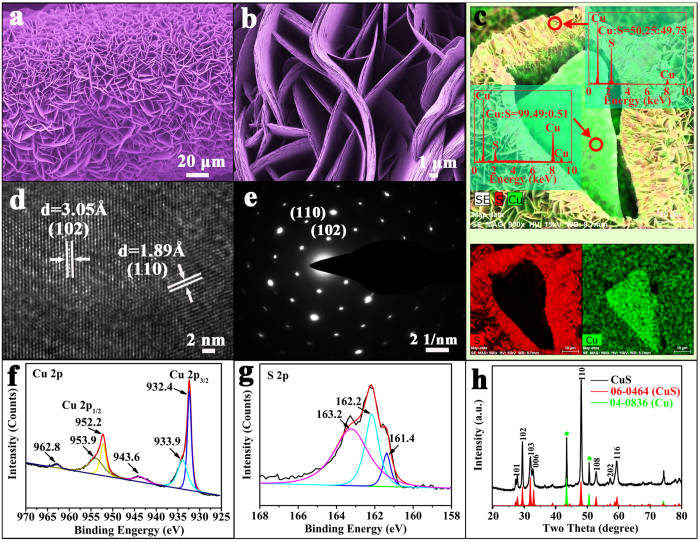
Characterization of Cu@CuS that obtained from the first reaction. (**a**,**b**) SEM images in low and high magnification, (**c**) EDX spectra and EDX elemental mappings for the element distribution, (**d**,**e**) HRTEM image and SAED pattern, (**f**,**g**) high resolution XPS spectra of Cu 2p and S 2p, (**h**) XRD pattern.

Cu@CuS catalyst was constructed by growing CuS nanosheets on copper foam. The low-magnification SEM image (Fig. 1a) shows that the as-prepared Cu@CuS consists of uniform curly microsheets with an average thickness of 1 μm. These microsheets are composed of a mass of nanosheets (Fig. 1b) and provide a wide growth space for the secondary reaction.

The EDX and EDX elemental mapping spectras of the external CuS nanosheets and internal copper framework are shown in Fig. 1c. These spectras can clearly indicate the distribution of CuS nanosheets and copper foam in the Cu@CuS, only Cu and S are found in both external nansheets and internal framework. Nevertheless, the elemental ratio of Cu and S significantly differs between the external nanosheets and internal framework. The elemental ratio of Cu and S in external CuS and internal framework are 50.25:49.75 (close to 1:1) and 99.49: 0.51, respectively. Therefore, the resultant of the first reaction is a mixture of unreacted Cu and CuS nanosheets, which provide a copper template for the secondary reaction.

HRTEM image was obtained to illustrate the geometrical structure of CuS nanosheets (Fig. 1d). The ordered lattice fringes clearly reveal the high crystallinity of the CuS nanosheets, and the distance of the adjacent lattice fringes are 3.05 Å and 1.89 Å, which are consistent to the (102) and (110) planes of CuS crystal (JCPDS no. 06–0464). The corresponding selected area electron diffraction (SAED) pattern (Fig. 1e) confirms the CuS nanosheets are single crystal with high crystallinity. The lattice spacings (d_102_ = 0.305 nm and d_110_ = 0.189 nm) can be indexed to the covellite phase of CuS. Thus, the CuS nanosheets grown on copper template are in pure phase.

XPS analysis was performed to investigate the chemical binding states of the product obtained from the first reaction. The high-resolution XPS spectrum of Cu in the 2p region for CuS nanosheets (Fig. 1f) was convoluted into two spin–orbit doublets and two shakeup satellites. The first doublet at 932.4 and 952.2 eV^38^, and the second at 933.9 and 953.9 eV^39^,^40^, were assigned to Cu 2p_3/2_ and Cu 2p_1/2_, respectively, and all belong to Cu (II). The satellites peaks present at 943.6 and 962.8 eV. Figure 1g shows the corresponding XPS spectrum of S 2p for CuS nanosheets. The S 2p spectrum at 163.2 eV^38^ and 162.2 eV^41^ can be assigned to S 2p_1/2_, and the peak at 161.4 eV^41^ belonged to S 2p_3/2_. The XPS spectra further indicate that the as-synthesized product is a pure CuS phase. The XRD pattern of Cu@CuS is shown in Fig. 1h. All the primary diffraction peaks of the curve are consistent with the standard card of CuS (JCPDS no. 06–0464) and Cu (JCPDS no. 04–0836). The diffraction peaks at 2θ = 27.68°, 29.28°, 31.79°, 32.82°, 47.95°, 52.71°, and 59.34° can be assigned to those of the planes (101), (102), (103), (006), (110), (108), and (116), respectively. In addition, two peaks of the Cu phase indicate that the copper foam is not completely reacted, which agrees with the aforementioned conclusion.

**Figure 2:**
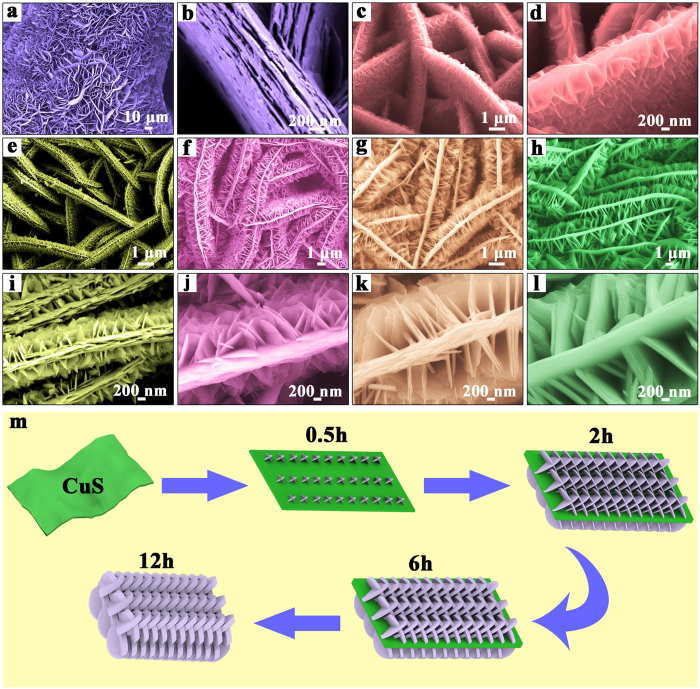
(**a**,**b**) SEM images in low and high magnification of Cu@CuS that obtained from the first reaction, SEM images in low and high magnification of (**c**,**d**) Cu@CuS-0.5, (**e,i**) Cu@CuS-1, (**f**,**j**) Cu@CuS-2, (**g**,**k**) Cu@CuS-4, (**h**,**l**) Cu@CuS-6 that obtained from the secondary reaction for 0.5 h, 1 h, 2 h, 4 h and 6 h, respectively, (**m**) growth schematic diagram of 3D hierarchical branched structure CuS nanosheet arrays during the secondary reaction.

The secondary reaction was conducted using Cu@CuS as precursor to investigate the crystal growth direction through the method similar to the first reaction. Reaction time has a significant influence on the formation of Cu@CuS nanosheet arrays with 3D hierarchical branched structure. A time-dependent experiment with 0.5, 1, 2, 4, 6, 8, 10, 12 h were conducted to obtain CuS nanosheet arrays with 3D hierarchical branched structure. The corresponding SEM images of Cu@CuS-0.5, Cu@CuS-1, Cu@CuS-2, Cu@CuS-4, Cu@CuS-6 are shown in Fig. 2c–i, and Cu@CuS-8, Cu@CuS-10 and Cu@CuS-12 are shown in Figure S1. The morphology of the as-synthesized resultants is considerably changed during the secondary reaction. Specifically, the morphology of Cu@CuS nanosheets with 2D curly structure (Fig. 2a,b) changes to Cu@CuS-0.5 nanosheet arrays with 3D hierarchical branched structure (Fig. 2c,d). The effect of reaction temperature on the structure of materials was also studied, the SEM images of materials for the secondary reaction under different temperature for 2 h: (a) 60 °C, (b) 80 °C, (c) 120 °C, (d) 140 °C were showed in the Figure S2. In these samples, a stack of uniform 3D hierarchical structure with the relative thin nanosheets that consist of the first nanosheets (average thickness of 100 nm) and the secondary nanosheets (thickness, approximately 18 nm, Fig. 2j) is presented perfectly in Cu@CuS-2. This structure would increase the specific surface area to provide sufficient dispersed active sites as a catalyst.

**Figure 3:**
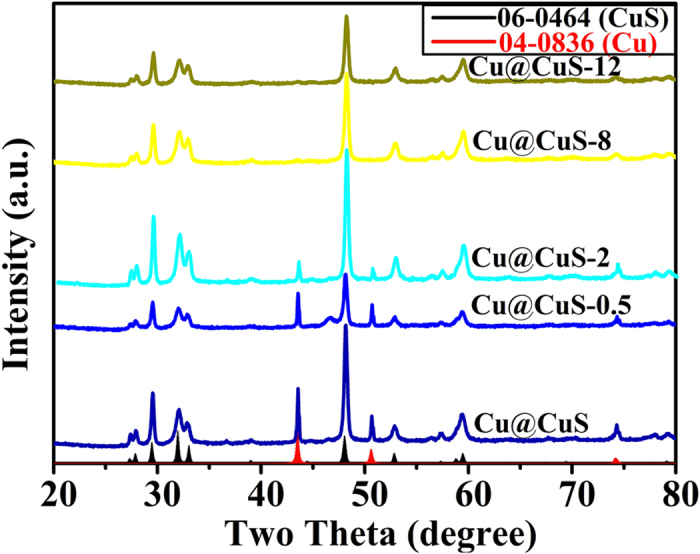
XRD patterns of Cu@CuS of the first reaction and Cu@CuS-0.5, Cu@CuS-2, Cu@CuS-8, Cu@CuS-12 that obtained from the secondary reaction for 0.5 h, 2 h, 8 h and 12 h, respectively.

A growth schematic diagram of the Cu@CuS nanosheet arrays with 3D hierarchical branched structure is shown in Fig. 2m to clearly illustrate the morphology evolution. The secondary nanosheets are generated on the surface of the Cu@CuS nanosheets obtained from the first reaction and form the 3D hierarchical branched structure. Generally, the reaction sites on the edge are more active than that on the surface of Cu@CuS nanosheets. However, during the secondary reaction, the S is dispersed uniformly in the solution, and sufficient Cu exists in the interior of the Cu@CuS template. The free Cu^2+^ originating from copper foam would react immediately with the S, and the secondary reaction is conducted along the surface of the first nanosheets nearest to the copper foam, hence the principle of proximity. Furthermore, the newly formed nanosheets grow larger with increasing reaction time because they contain active sites similar to the first nanosheets. On the contrary, crystal growth direction is limited because of the shrunken space between the secondary nanosheets. Consequently, the secondary reaction only occurred around the secondary nanosheets, and the secondary nanosheets thicken gradually until the branched structure disappeared completely after 12 h. The corresponding XRD patterns of Cu@CuS, Cu@CuS-0.5, Cu@CuS-2, Cu@CuS-8 and Cu@CuS-12 are shown in Fig. 3. The strong and sharp diffraction peaks of Cu@CuS-2 in the curve are the strongest, which indicates that as-synthesized Cu@CuS-2 is well crystallized. Cu phase disappeared with the increasing reaction time, which illustrates that the copper foam is completely transformed into CuS.

The influence for the amount of sulfur powder on morphology was explored in the secondary reaction. The amount of sulfur powder in the Cu@CuS-2-1 and Cu@CuS-2-2 were adjusted to 1 and 3 mmol, respectively, and the corresponding SEM images are shown in Figure S3. The high magnification SEM images in the Figure S3b and S3d present disordered secondary structure and multiple complex morphologies appearing on the 3D framework with increasing amount of sulfur powder (Figure S3c). Therefore, appropriate amount of sulfur powder is important for the formation of 3D hierarchical branched structure.

**Figure 4:**
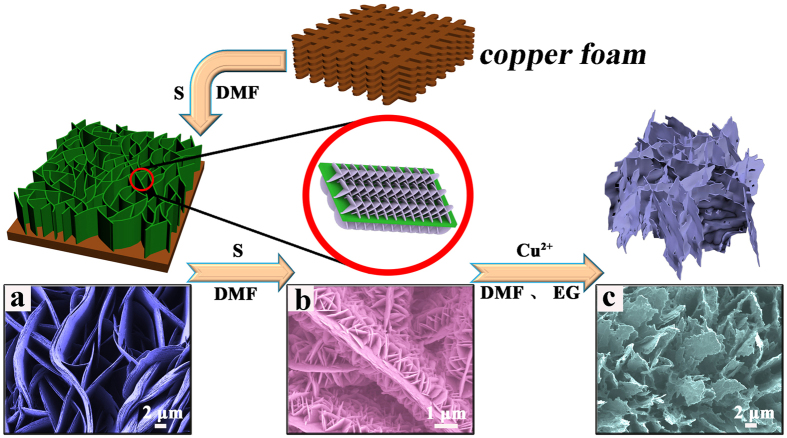
Schematic diagrams of the structure change of the products (**a**) Cu@CuS of the first reaction, (**b**) Cu@CuS-2 of the secondary reaction for 2 h and (**c**) CuS@Cu_9_S_5_-1 of cation exchange with DMF and EG as solvents, respectively.

A schematic diagram of the transformation from copper foam to cation exchange is shown in Fig. 4. The structure of each reaction significantly change, which considerably influence the catalytic property. In this process, the Cu@CuS-2 nanosheet arrays with 3D hierarchical branched structure as template can provide a large specific surface area for cation exchange, the Cu^2+^ of Cu(NO_3_)_2_•3H_2_O is dispersed uniformly in the solution to replace the unreacted copper foam in the Cu@CuS-2, and the exchanged resultant may grow along the surface of Cu@CuS-2 nanosheets because of the similar crystal growth direction principle with the secondary reaction. In this process, a few amount of Cu^2+^ maybe generated from the CuS of Cu@CuS-2 and may participate in the cation exchange, thereby leading to significant morphological changes in CuS@Cu_9_S_5_-1.

**Figure 5:**
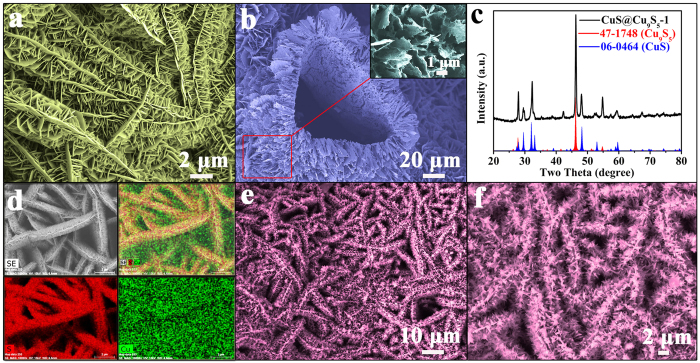
SEM images of (**a**) Cu@CuS-2-3 that obtained from the third reaction at 100 °C for 2 h, (**b**) CuS@Cu_9_S_5_-1 that obtained by cation exchange, (**c**) XRD pattern of CuS@Cu_9_S_5_-1, (**d**) EDX elemental mapping images for Cu and S of Cu@CuS-2, (**e**,**f**) SEM images in low and high magnification of Cu@CuS-2 after degradation of MB.

Sulfur powder, a vital influencing factor, was introduced into the third reaction, and then the Cu@CuS-2-3 (Fig. 5a) with 3D hierarchical branched structure was obtained using Cu@CuS-2 as template at 100 °C for 2 h. This structure was similar to that of Cu@CuS-2, which illustrates that 3D hierarchical branched structure is likely to stabilize in the secondary reaction and the crystal growth direction is well controlled by the facial consecutive reaction. Copper is the other crucial factor for the formation of 3D hierarchical branched structure. Furthermore, industrial wastewater possibly has abundant Cu^2+^ in the process of printing and dyeing. Thus, Cu^2+^ as considerable candidate was selected to replace the unreacted copper foam of Cu@CuS-2 to further investigate crystal growth direction and the morphology, and the SEM image of the exchanged resultant of CuS@Cu_9_S_5_-1 is shown in Fig. 5b, in which Cu@CuS-2 nanosheet arrays with 3D hierarchical branched structure change into CuS@Cu_9_S_5_-1 with 2D leaf-like structure, and copper foam was exchanged completely. The XRD pattern in Fig. 5c shows that all the diffraction peaks in the curve are consistent with the standard card of CuS (JCPDS no. 06-0464) and Cu_9_S_5_ (JCPDS no. 47–1748). This result indicates that cation exchange occured between the Cu of Cu@CuS-2 and Cu^2+^ of Cu(NO_3_)_2_•3H_2_O. Hence, cation exchange can also happen in the other solvent, such as pure DMF. The corresponding SEM images of the as-obtained CuS@Cu_9_S_5_-2 are shown in Figure S4, in which the morphology differs from that of CuS@Cu_9_S_5_-1. The XRD pattern of CuS@Cu_9_S_5_-2 shown in Figure S6 is similar to that of CuS@ Cu_9_S_5_-1, which indicates that the components of the exchanged resultant remain similarity even in different solvents. EDS mapping in Fig. 5d reveals that the Cu and S in the Cu@CuS-2 nanosheet arrays are distributed homogeneously. This indicates that Cu@CuS-2 with 3D hierarchical branched structure possesses uniform nanosheets with large aperture ratio, which could provide sufficient reaction active sites as catalyst.

**Figure 6:**
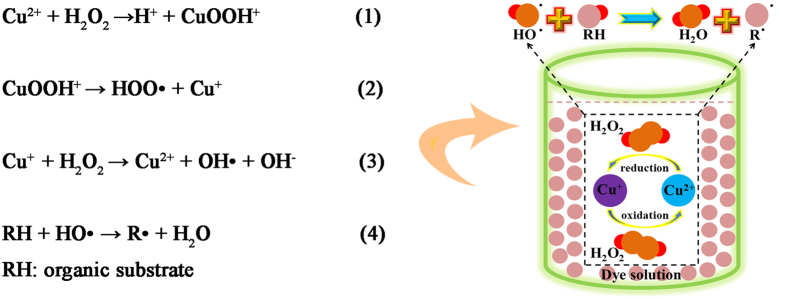
The degradation mechanism diagram of the CuS catalysts in the dark environment.

These kinds of CuS materials with distinct morphorlogy were used as catalysts for absorption and degradation of MB molecules. The related experiments were conducted with adding of H_2_O_2_. The degradation mechanism diagram for a series of CuS catalysts in the dark environment is summarized in the pathway that shown in the Fig. 6. In this degradation process, H_2_O_2_ yielded largely reactive hydroxyl radicals, which can oxidize the MB molecules into smaller molecules (CO_2_ and H_2_O). A brief description of the reaction mechanism is further described in the following equation^42^ in the Fig. 6.

**Figure 7:**
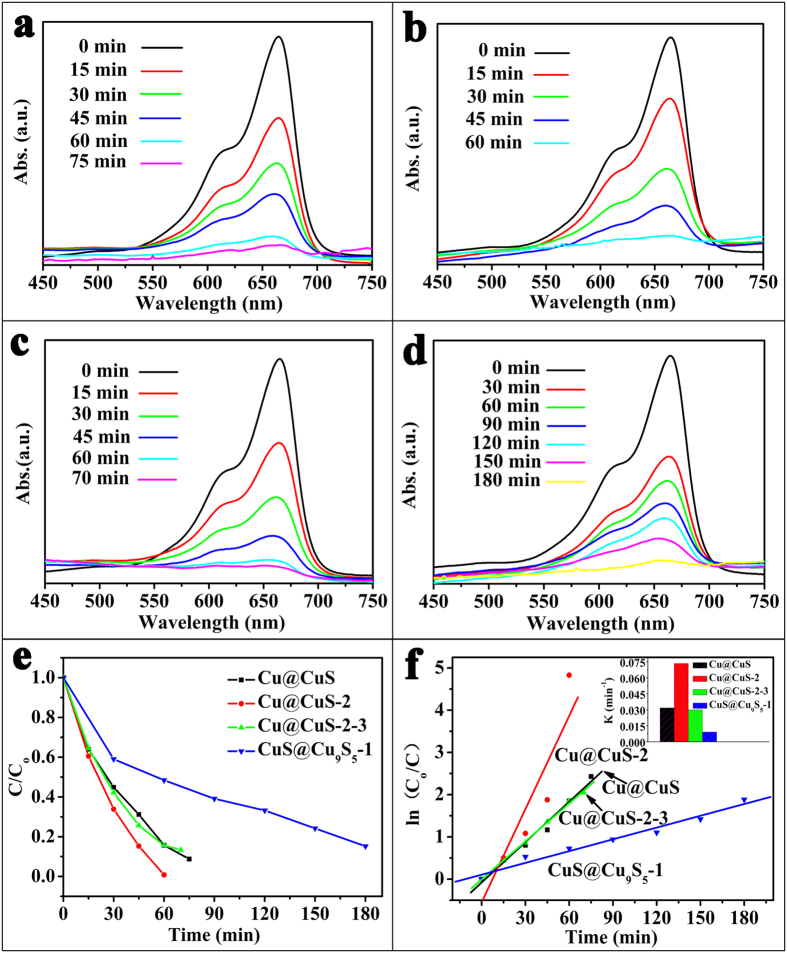
Changes in the UV-vis spectra during the removal of MB by (**a**) Cu@CuS, (**b**) Cu@CuS-2, (**c**) Cu@CuS-2-3, (**d**) CuS@Cu_9_S_5_-1, (**e**) Degeneration curves for MB by different samples, (**f**) Kinetics study for the degradation of MB: the inset shows the first order rate constant for Cu@CuS, Cu@CuS-2, Cu@CuS-2-3, CuS@Cu_9_S_5_-1.

The catalytic properties of the materials are associated with the amount of hydroxyl radicals. The degradation rate of H_2_O_2_ alone for the dye solutions without the assistance of the catalyst is slow^32^. The use of only the CuS product with 3D framework as catalyst also contribute few to the catalytic process. The catalytic properties for MB of as-synthesized samples are investigated by the UV-vis absorption spectras and shown in Fig. 7a–d. Samples exhibit catalytic activity with the efficient catalysis of H_2_O_2_ to release hydroxyl radicals (•OH) and degrade the MB in the dark environment. The decoloring degree of aqueous MB by Cu@CuS in Fig. 7e reached 36%, 55.2%, 68.8%, and 84.3% after 15, 30, 45, 60 min, respectively. After 75 min, the decoloring degree achieves 91.2%. Amazingly, the decoloring degree for MB by Cu@CuS-2 reached 99.2% after 60 min, this good catalytic performance can attribute to the unique 3D hierarchical branched structure of Cu@CuS-2, which provides high specific surface area as catalyst that just as we aforementioned prediction. However, the decoloring degree for MB by Cu@CuS-2-3 and CuS@Cu_9_S_5_-1 reached 84.3% and 51.6% after 60 min, respectively. Compared with Cu@CuS-2, the crystal growth direction of Cu@CuS-2-3 unchanged, and the crystal continue to grow along with the original secondary nanosheet. Therefore, the spacing between the secondary nanosheets decreased during the third reaction, resulting in the diminished specific surface area effects the catalytic performance seriously. For CuS@Cu_9_S_5_-1, the catalytic performance indicates Cu@CuS-2 still has the capacity to degrade dye solution after cation exchange, despite it shows a low decoloring degree. To further study the degradation kinetics, the first order rate constant of Cu@CuS, Cu@CuS-2, Cu@CuS-2-3 and CuS@Cu_9_S_5_-1 for degradation of MB (Fig. 7f) were calculated to be 0.032 min^−1^, 0.074 min^−1^, 0.03 min^−1^, 0.009 min^−1^, respectively. It obviously seen that the decoloring rate of Cu@CuS-2 is hihger than that of other samples. Delightfully, the SEM images in low and high magnification of Cu@CuS-2 after degradation of MB (Fig. 5e,f) show that it maintains the 3D hierarchical branched structure. And the first order rate constant is described as following^43^:


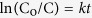


where *k* is the apparent rate constant, C_o_ is the original concentration of MB or MB@RB. and C is equilibrium concentration of MB or MB@RB at the relative reaction time.

**Figure 8:**
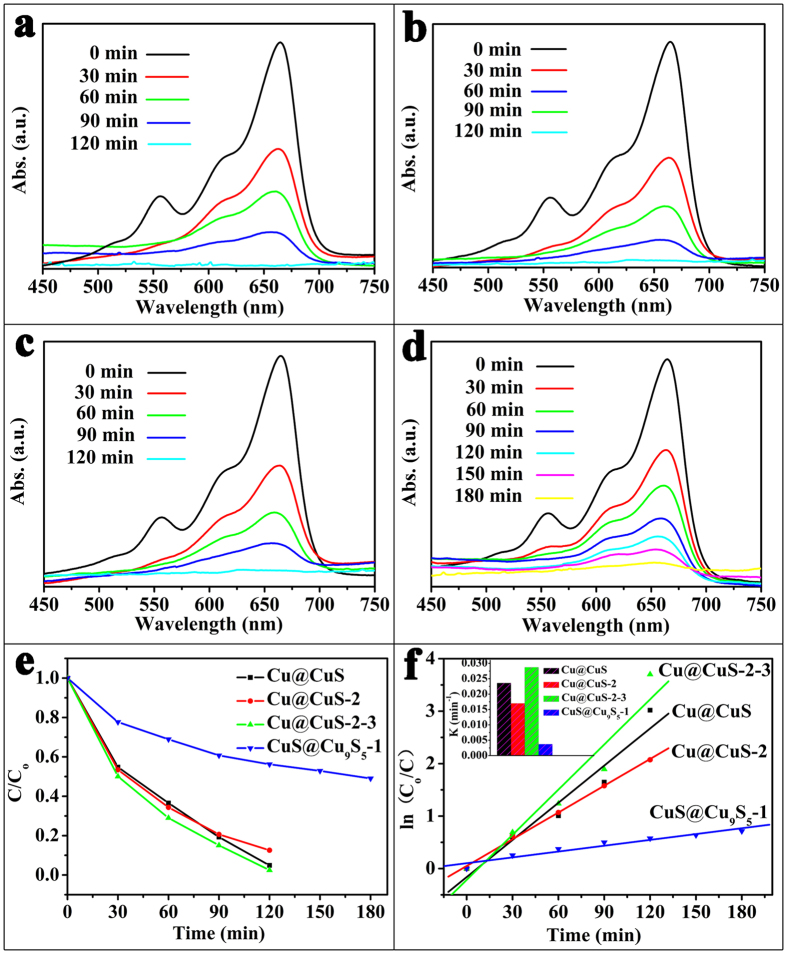
Changes in the UV-vis spectra during the removal of the mixed solution of MB@RB by (**a**) Cu@CuS, (**b**) Cu@CuS-2, (**c**) Cu@CuS-2-3, (**d**) CuS@Cu_9_S_5_-1, (**e**) Degeneration curves for the mixed solution of MB@RB by different samples, (**f**) Kinetics study for the degradation of the mixed solution of MB@RB: the inset shows the first order rate constant for Cu@CuS, Cu@CuS-2, Cu@CuS-2-3, CuS@Cu_9_S_5_-1.

This kind of catalyst can not only degrade a single organic dye, such as MB and RB, but can also degrade the mixed solution composed of different kinds of dyes. The catalytic properties for the mixed solution of MB@RB of as-synthesized samples are investigated by the UV-vis absorption spectras and shown in Fig. 8a–d. The decoloring degree of the mixed solution of MB@RB by Cu@CuS in Fig. 8e reached 45.2%, 63.4%, 90.7%, and 95.1% after 30, 60, 90, 120 min, respectively. Nevertheless, the decoloring degree are 87.4% of Cu@CuS-2, 97.5% of Cu@CuS-2-3 and 43.8% of CuS@Cu_9_S_5_-1 under the same condition to degrade the mixed solution of MB@RB after 120 min. The first order rate constant of Cu@CuS, Cu@CuS-2, Cu@CuS-2-3 and CuS@Cu_9_S_5_-1 for degradation of the mixed solution of MB@RB (Fig. 8f) were calculated to be 0.024 min^−1^, 0.017 min^−1^, 0.029 min^−1^, 0.004 min^−1^, respectively. However, the degradation efficiency of the mixed solution of MB@RB by Cu@CuS-2 is lower than that of Cu@CuS, Cu@CuS-2–3 because of the complicated functional mechanism of the mixed solution of MB@RB that we can’t corfirm. The SEM images of Cu@CuS-2 after degradation of mixed solution of MB and RB are shown in Figure S5, they also maintain the 3D hierarchical branched structure.

The stable structure of Cu@CuS-2 may cause the easier recycling of these catalysts than that of powder materials, such as ZnS^44^, TiO_2_^45^, and WO_3_^46^. This result demonstrates that the materials with 3D hierarchical branched structure have a promising potential as an excellent recyclable catalyst. Meanwhile, cation exchange occurring in different solvents indicates that Cu@CuS-2 with 3D hierarchical branched structure has a significant role in effective absorption of Cu^2+^ from wastewater. Moreover, the degradation of the mixed solution of MB@RB can be beneficial to the study of the composite system of dye solution and complex environment, which further reveals the catalytic panorama of the industrial wastewater.

## Conclusion

The crystal growth direction of CuS were effectively controlled by a simple *in situ* consecutive reaction using copper foam as template and raw material, and the structures are different for each reaction. Cation exchange between the same element occurred by using the Cu@CuS-2 of the secondary reaction as precursor. The growth mechanism of 3D hierarchical branched structure of the secondary reaction was mainly determined by the reaction time. The as-synthesized Cu@CuS-2 nanosheet arrays with 3D hierarchical branched structure were grown on the surface of the copper framework firmly and the stable 3D hierarchical branched structure was certified from the third reaction. This structure provided a large specific surface area and sufficient active sites as catalyst. CuS@Cu_9_S_5_-1 with leaf-like structure was also synthesized by Cu^2+^ exchange method with Cu@CuS-2 as precursor. In this process, the morphological change revealed that Cu@CuS-2 with 3D hierarchical branched structure can not only degrade an organic dye effectively but also absorb Cu^2+^ in industrial wastewater. Therefore, this *in situ* consecutive reaction has a potentail meaning in controlling and designing the crystal growth of inorganic material.

## Additional Information

**How to cite this article**: Zhang, X. *et al*. Consecutive Reaction to Construct Hierarchical Nanocrystalline CuS “Branch” with Tunable Catalysis Properties. *Sci. Rep.* **6**, 30604; doi: 10.1038/srep30604 (2016).

## Supplementary Material

Supplementary Information

## References

[b1] Zhang F. L. . Hierarchical Nanowire Arrays as Three-Dimensional Fractal Nanobiointerfaces for High Efficient Capture of Cancer Cells. Nano Lett. 16, 766–772 (2016). 26673032 10.1021/acs.nanolett.5b04731

[b2] Yang H. C., Zhang Y. J., Hu F. & Wang Q. B. Urchin-like CoP Nanocrystals as Hydrogen Evolution Reaction and Oxygen Reduction Reaction Dual-Electrocatalyst with Superior Stability. Nano Lett. 15, 7616–7620 (2015). 26474359 10.1021/acs.nanolett.5b03446

[b3] Wei W. . Preparation of Hierarchical Hollow CaCO_3_ Particles and the Application as Anticancer Drug Carrier. J. Am. Chem. Soc. 130, 15808–15810 (2008). 18980322 10.1021/ja8039585

[b4] Shi Y. T. . Ultrarapid Sonochemical Synthesis of ZnO Hierarchical Structures: From Fundamental Research to High Efficiencies up to 6.42% for Quasi-Solid Dye-Sensitized Solar Cells. Chem. Mater. 25, 1000–1012 (2013).

[b5] Wang H. K. & Rogach A. L. Hierarchical SnO_2_ Nanostructures: Recent Advances in Design, Synthesis, and Applications. Chem. Mater. 26, 123–133 (2014).

[b6] Gao Y. . Double Metal Ions Synergistic Effect in Hierarchical Multiple Sulfide Microflowers for Enhanced Supercapacitor Performance. ACS Appl. Mater. Interfaces 7, 4311–4319 (2015). 25625946 10.1021/am508747m

[b7] Sun K. . Solution Synthesis of Large-Scale, High-Sensitivity ZnO/Si Hierarchical Nanoheterostructure Photodetectors. J. Am. Chem. Soc. 132, 15465–15467 (2010). 20949970 10.1021/ja1038424

[b8] Sheng X., He D. Q., Yang J., Zhu K. & Feng X. J. Oriented Assembled TiO_2_ Hierarchical Nanowire Arrays with Fast Electron Transport Properties. Nano Lett. 14, 1848–1852 (2014). 24628675 10.1021/nl4046262

[b9] Li Y. Y., Li Z. S. & Shen P. K. Simultaneous Formation of Ultrahigh Surface Area and Three-Dimensional Hierarchical Porous Graphene-Like Networks for Fast and Highly Stable Supercapacitors. Adv. Mater. 25, 2474–2480 (2013). 23495046 10.1002/adma.201205332

[b10] Gao H. L. . Macroscopic Free-Standing Hierarchical 3D Architectures Assembled from Silver Nanowires by Ice Templating. Angew. Chem. Int. Ed. 53, 4561–4566 (2014). 10.1002/anie.201400457 24683064

[b11] Han R. . Geometry-Assisted Three-Dimensional Superlocalization Imaging of Single-Molecule Catalysis on Modular Multilayer Nanocatalysts. Angew. Chem. Int. Ed. 126, 13079–13083 (2014). 10.1002/anie.201407140 25257929

[b12] Jiang Y. . Boosting the Open Circuit Voltage and Fill Factor of QDSSCs Using Hierarchically Assembled ITO@Cu_2_S Nanowire Array Counter Electrodes. Nano Lett. 15, 3088–3095 (2015). 25929671 10.1021/acs.nanolett.5b00096

[b13] Zhuang J. L. . Synthesis of a New Copper-Azobenzene Dicarboxylate Framework in the Form of Hierarchical Bulk Solids and Thin Films without and with Patterning. Chem. Mater. 23, 5366–5374 (2011).

[b14] Lao J. Y., Wen J. G. & Ren Z. F. Hierarchical ZnO Nanostructures. Nano Lett. 2, 1287–1291 (2002).

[b15] Ji M., Cai J. G., Ma Y. R. & Qi L. M. Controlled Growth of Ferrihydrite Branched Nanosheet Arrays and Their Transformation to Hematite Nanosheet Arrays for Photoelectrochemical Water Splitting. ACS Appl. Mater. Interfaces 8, 3651–3660 (2016). 26517010 10.1021/acsami.5b08116

[b16] Zhang J., Yu J. G., Zhang Y. M., Li Q. & Gong J. R. Visible Light Photocatalytic H_2_-Production Activity of CuS/ZnS Porous Nanosheets Based on Photoinduced Interfacial Charge Transfer. Nano Lett. 11, 4774–4779 (2011). 21981013 10.1021/nl202587b

[b17] Zhang W. L. . Heterostructures of CuS nanoparticle/ZnO nanorod arrays on carbon fibers with improved visible and solar light photocatalytic properties. J. Mater. Chem. A. 3, 7304–7313 (2015).

[b18] Li Y. X. . Hierarchical ZnS-In_2_S_3_-CuS Nanospheres with Nanoporous Structure: Facile Synthesis, Growth Mechanism, and Excellent Photocatalytic Activity. Adv. Funct. Mater. 20, 3390–3398 (2010).

[b19] Li L. L., Samakrishna S. . Controlled Growth of CuS on Electrospun Carbon Nanofibers as an Efficient Counter Electrode for Quantum Dot-Sensitized Solar Cells. J. Phys. Chem. C 118, 16526–16535 (2014).

[b20] Ye M. D. . *In situ* growth of CuS and Cu_1.8_S nanosheet arrays as efficient counter electrodes for quantum dotsensitized solar cells. J. Mater. Chem. A. 3, 9595–9600 (2015).

[b21] Yang Z. S., Chen C. Y., Liu C. W., Li C. L. & Chang H. T. Quantum Dot–Sensitized Solar Cells Featuring CuS/CoS Electrodes Provide 4.1% Efficiency. Adv. Energy Mater. 1, 259–264 (2011).

[b22] Tian Q. W. . Hydrophilic Flower-Like CuS Superstructures as an Efficient 980 nm Laser-Driven Photothermal Agent for Ablation of Cancer Cells. Adv. Mater. 23, 3542–3547 (2011). 21735487 10.1002/adma.201101295

[b23] Chen F. . *In Vivo* Tumor Vasculature Targeting of CuS@MSN Based Theranostic Nanomedicine. ACS Nano 9, 3926–3934 (2015). 25843647 10.1021/nn507241v PMC4414921

[b24] Riedinger A. . Post-Synthesis Incorporation of ^64^Cu in CuS Nanocrystals to Radiolabel Photothermal Probes: A Feasible Approach for Clinics. J. Am. Chem. Soc. 137, 15145–15151 (2015). 26551614 10.1021/jacs.5b07973

[b25] Peng H. . High-performance supercapacitor based on multi-structural CuS@polypyrrole composites prepared by *in situ* oxidative polymerization. J. Mater. Chem. A. 2, 3303–3307 (2014).

[b26] Zhang J. . Solvothermal Synthesis of Three-Dimensional Hierarchical CuS Microspheres from a Cu-Based Ionic Liquid Precursor for High Performance Asymmetric Supercapacitors. ACS Appl. Mater. Interfaces 7, 21735–21744 (2015). 26371955 10.1021/acsami.5b04452

[b27] Ge L. . Ionic Liquid-Assisted Synthesis of CuS Nestlike Hollow Spheres Assembled by Microflakes Using an Oil–Water Interface Route. Cryst. Growth Des. 10, 1688–1692 (2010).

[b28] Nagarathinam M., Chen J. L. & Vittal J. V. From Self-Assembled Cu(II) Coordination Polymer to Shape-Controlled CuS Nanocrystals. Cryst. Growth Des. 9, 2457–2463 (2009).

[b29] Zhu H. T., Wang J. X. & Wu D. X. Fast Synthesis, Formation Mechanism, and Control of Shell Thickness of CuS Hollow Spheres. Inorg. Chem. 48, 7099–7104 (2009). 19585979 10.1021/ic900201p

[b30] He W. W. . Understanding the formation of CuS concave superstructures with peroxidase-like activity. Nanoscale 4, 3501–3506 (2012). 22552534 10.1039/c2nr30310h

[b31] Zhao B. . Synthesis of flower-like CuS hollow microspheres based on nanoflakes self-assembly and their microwave absorption properties. J. Mater. Chem. A. 3, 10345–10352 (2015).

[b32] Mi L. W. . Tunable properties induced by ion exchange in multilayer intertwined CuS microflowers with hierarchal structures. Nanoscale 5, 6589–6598 (2013). 23760635 10.1039/c3nr01438j

[b33] Wu X. . Synthesis of Hollow Cd_x_Zn_1−x_Se Nanoframes through the Selective Cation Exchange of Inorganic–Organic Hybrid ZnSe-Amine Nanoflakes with Cadmium Ions. Angew. Chem. Int. Ed. 51, 3211–3215 (2012). 10.1002/anie.201108098 22334529

[b34] Justo Y. . Less Is More. Cation Exchange and the Chemistry of the Nanocrystal Surface. ACS Nano 8, 7948–7957 (2014). 25090034 10.1021/nn5037812

[b35] Miszta K., Dorfs D., Genovese A., Kim M. R. & Manna L. Cation Exchange Reactions in Colloidal Branched Nanocrystals. ACS Nano 5, 7176–7183 (2011). 21809824 10.1021/nn201988w

[b36] Lubeck C. R., Han T. Y. J., Gash A. E., Satcher J. H. & Doyle F. M. Synthesis of Mesostructured Copper Sulfide by Cation Exchange and Liquid-Crystal Templating. Adv. Mater. 18, 781–784 (2006).

[b37] van der Stam W. . Luminescent CuInS_2_ Quantum Dots by Partial Cation Exchange in Cu_2−x_S Nanocrystals. Chem. Mater. 27, 621–628 (2015).

[b38] Liu P. B., Huang Y., Yan J., Yang Y. W. & Zhao Y. Construction of CuS Nanoflakes Vertically Aligned on Magnetically Decorated Graphene and Their Enhanced Microwave Absorption Properties. ACS Appl. Mater. Interfaces 8, 5536–5546 (2016). 26886765 10.1021/acsami.5b10511

[b39] Naghash A. R., Etsell T. H. & Xu S. XRD and XPS Study of Cu-Ni Interactions on Reduced Copper-Nickel-Aluminum Oxide Solid Solution Catalysts. Chem. Mater. 18, 2480–2488 (2006).

[b40] Chen S. S. . Oxidation Resistance of Graphene- Coated Cu and Cu/Ni Alloy. ACS nano 5, 1321–1327 (2011). 21275384 10.1021/nn103028d

[b41] Wei W. T. . Partial Ion-Exchange of Nickel-Sulfide-Derived Electrodes for High Performance Supercapacitors. Chem. Mater. 26, 3418–3426 (2014).

[b42] Rhadfi T. . Polyol-made Mn_3_O_4_ nanocrystals as efficient Fenton-like catalysts. Appl. Catal. A 386, 132–139 (2010).

[b43] Cao H. L. . High symmetric 18-facet polyhedron nanocrystals of Cu_7_S_4_ with a hollow nanocage. J. Am. Chem. Soc. 127, 16024–16025 (2005). 16287279 10.1021/ja055265y

[b44] Naghiloo S., Habibi-Yangjeh A. & Behboudnia M. Adsorption and photocatalytic degradation of methylene blue on Zn_1−x_ Cu_x_S nanoparticles prepared by a simple green method. Appl. Surf. Sci. 257, 2361–2366 (2011).

[b45] Moya A. . Oxygen vacancies and interfaces enhancing photocatalytic hydrogen production in mesoporous CNT/TiO_2_ hybrids. Appl. Catal. B. Environ. 179, 574–582 (2015).

[b46] Sayama K. . Highly active WO_3_ semiconductor photocatalyst prepared from amorphous peroxo-tungstic acid for the degradation of various organic compounds. Appl. Catal. B. Environ. 94, 150–157 (2010).

