# Supplementary material for: Consecutive Reaction to Construct Hierarchical Nanocrystalline CuS “Branch” with Tunable Catalysis Properties
Source: Sci Rep. 2016 Jul 28;6:30604. doi: 10.1038/srep30604 (PMC4964342; doi:10.1038/srep30604)
Supplement: Supplementary Information [file srep30604-s1.doc]

**Supporting Informafion**

**Consecutive Reaction to Construct Hierarchical Nanocrystalline CuS “Branch” With Tunable Catalysis Properties**

Xiangdan Zhang,^[a]^ Feifei Yang,^[b]^ Shizhong Cui,^[a]^ Wutao Wei,^[a]^ Weihua Chen^[b],*^ and Liwei Mi^[a],*^

^[a]^ Center for Advanced Materials Research, Zhongyuan University of Technology, Zhengzhou, Henan 450007, P. R. China.;

^[b]^ College of Chemistry and Molecular Engineering, Zhengzhou University, Zhengzhou, Henan 450001, P. R. China.

* Corresponding authors. Email: mlwzzu@163.com (Liwei Mi) and chenweih@zzu.edu.cn (Weihua Chen).


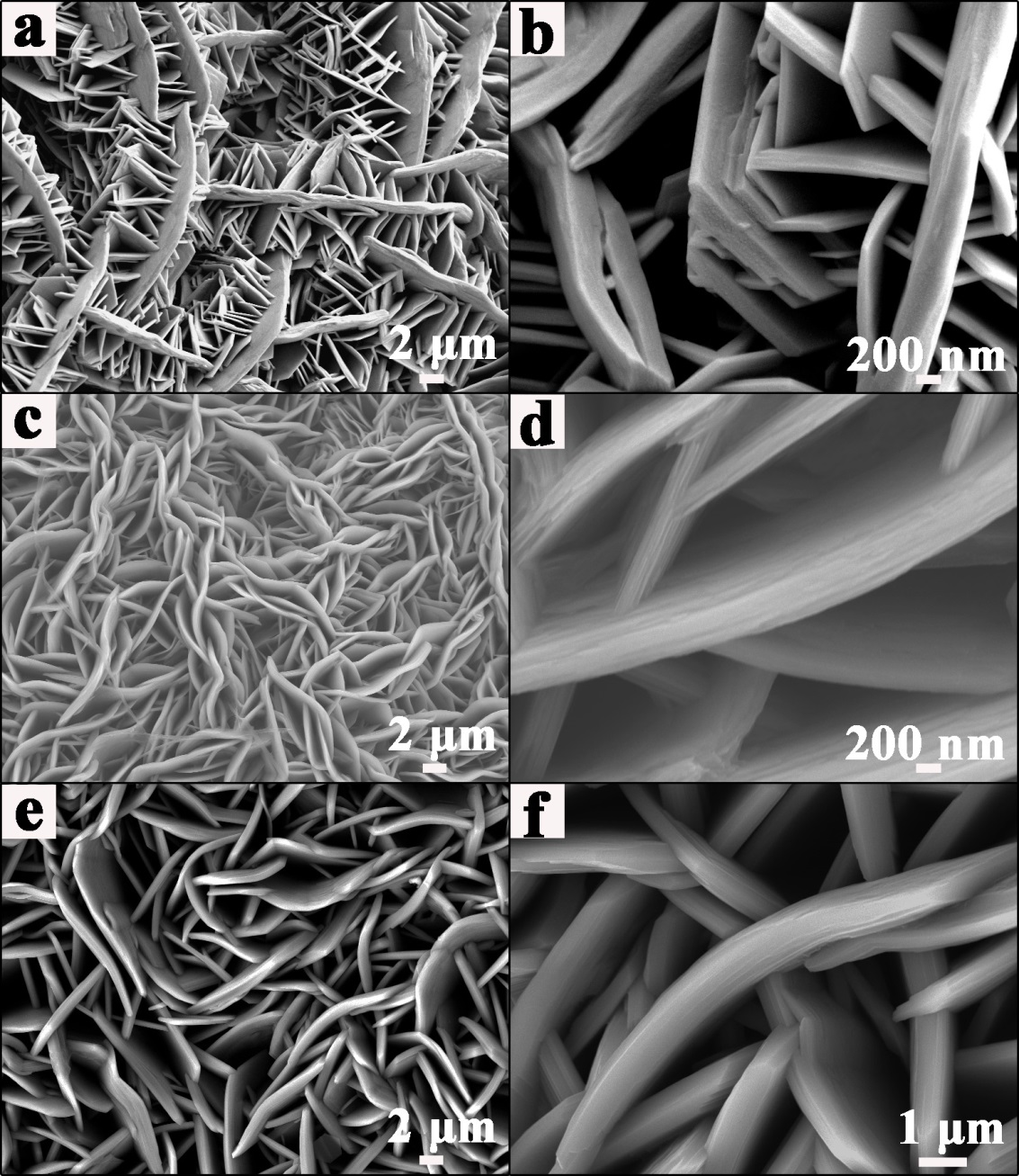


**Figure S1**. SEM images in low and high magnification of Cu@CuS-8 (a and b), Cu@CuS-10 (c and d) and Cu@CuS-12 (e and f).


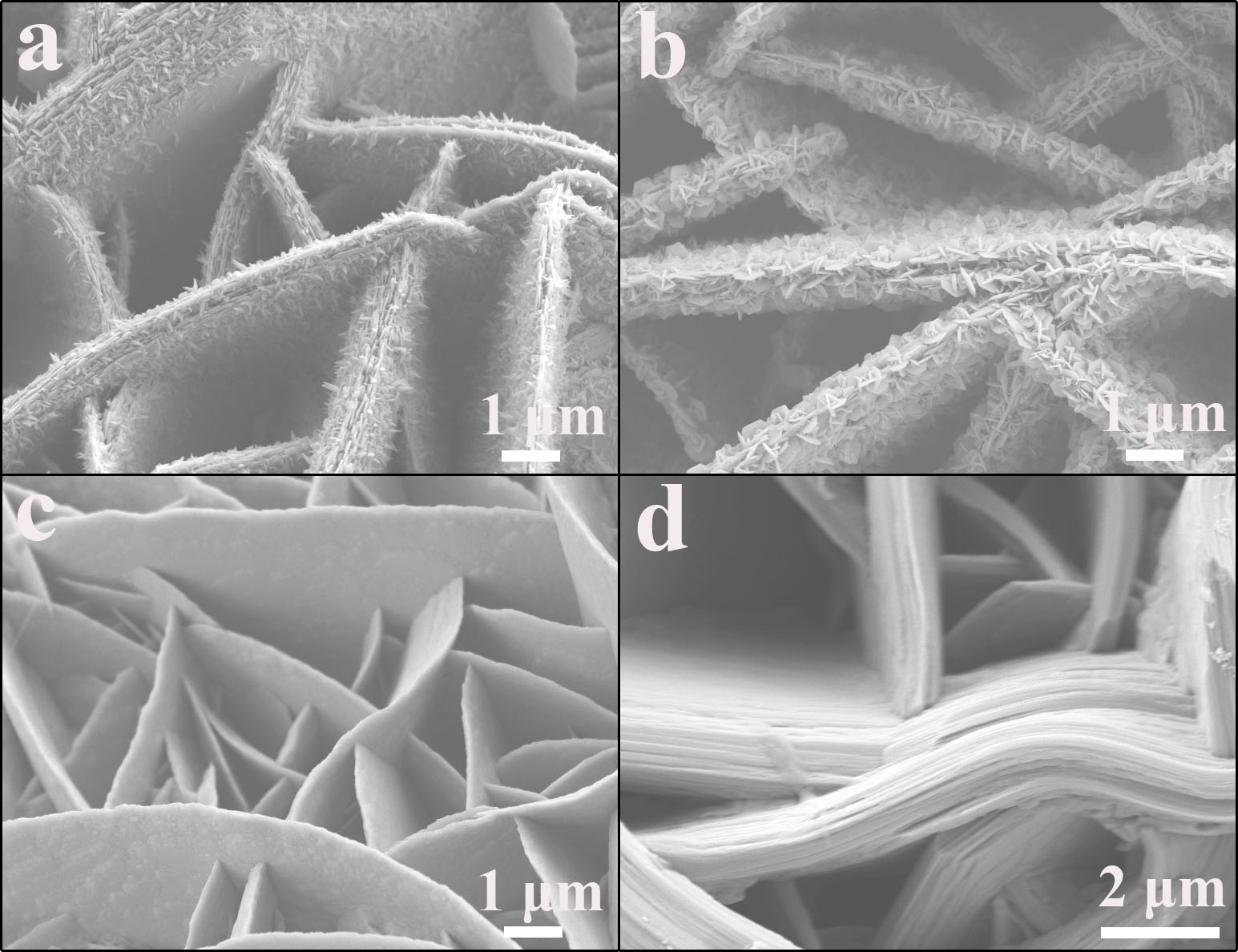


**Figure S2**. The SEM images of CuS materials for the secondary reaction under different temperature for 2 h: (a) 60 ^o^C; (b) 80 ^o^C; (c) 120 ^o^C; (d) 140 ^o^C.


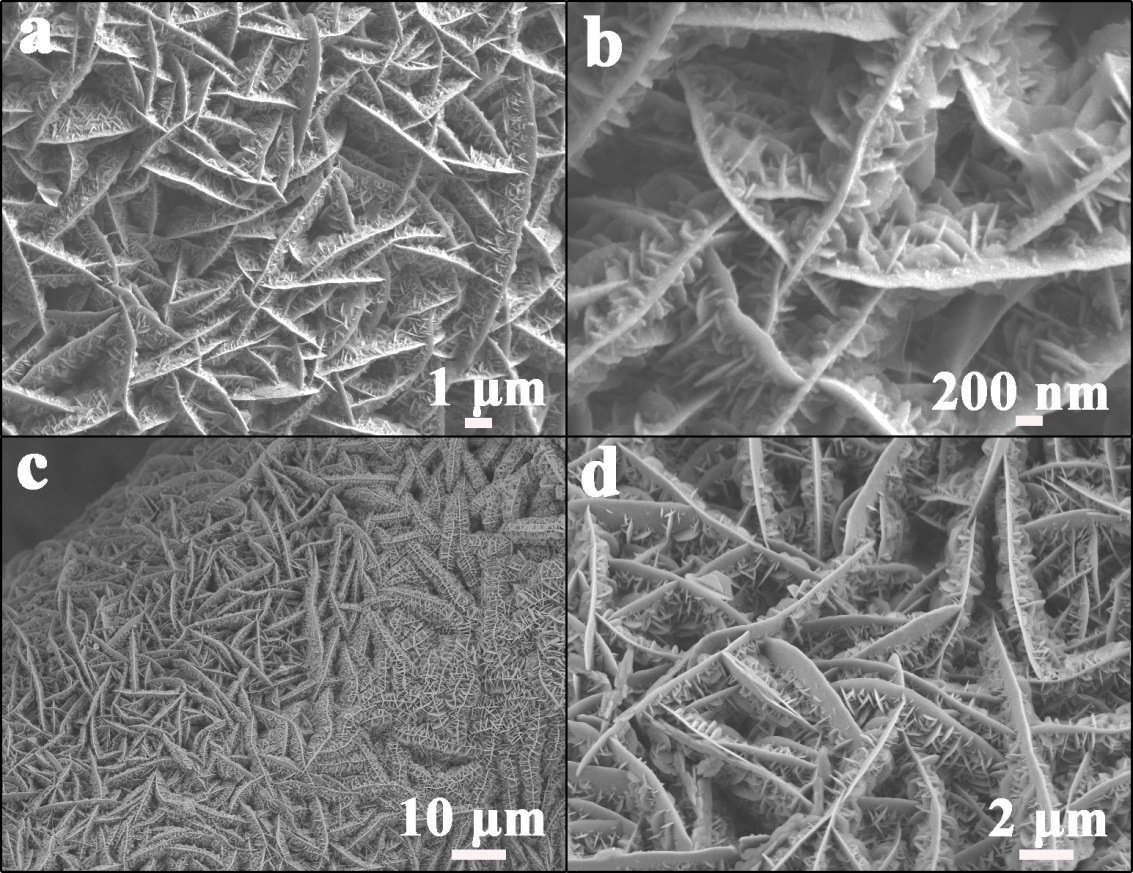


**Figure S3**. SEM images in low and high magnification of Cu@CuS-8 (a and b), Cu@CuS-10 (c and d)


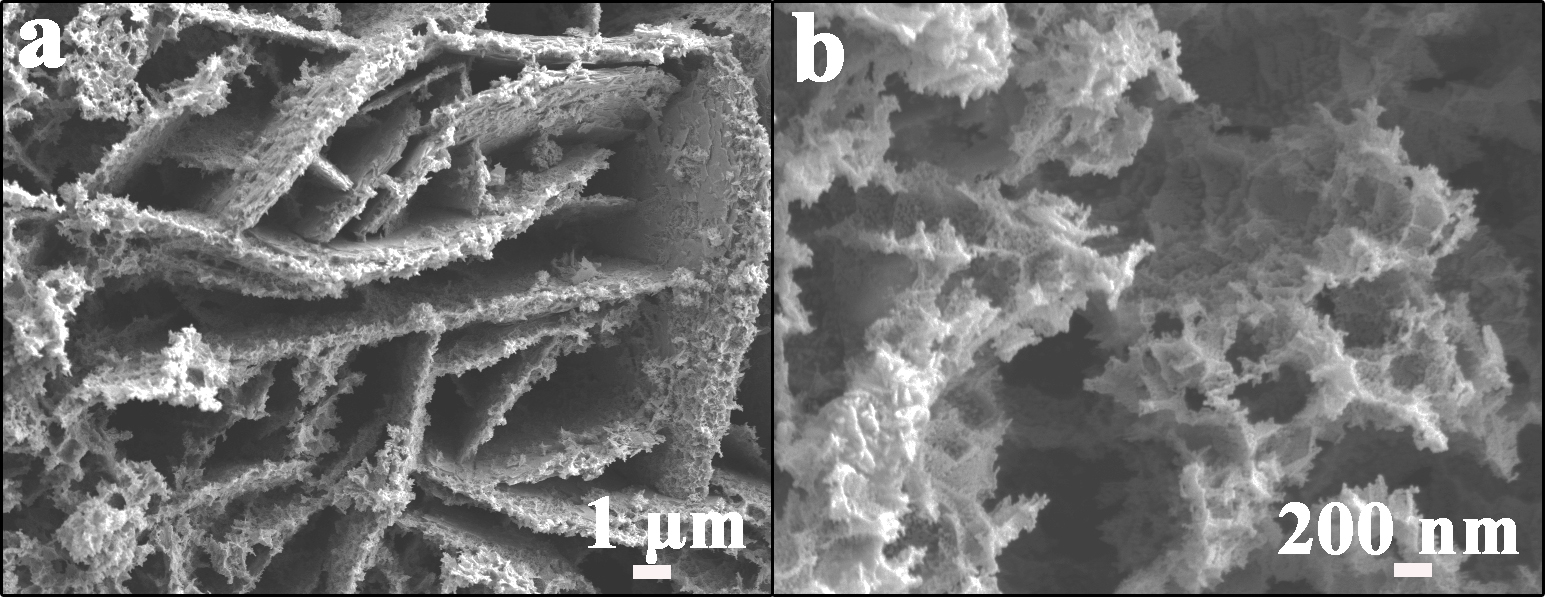


**Figure S4**. SEM images in low and high magnification of CuS@Cu_9_S_5_-2 (a and b)

**
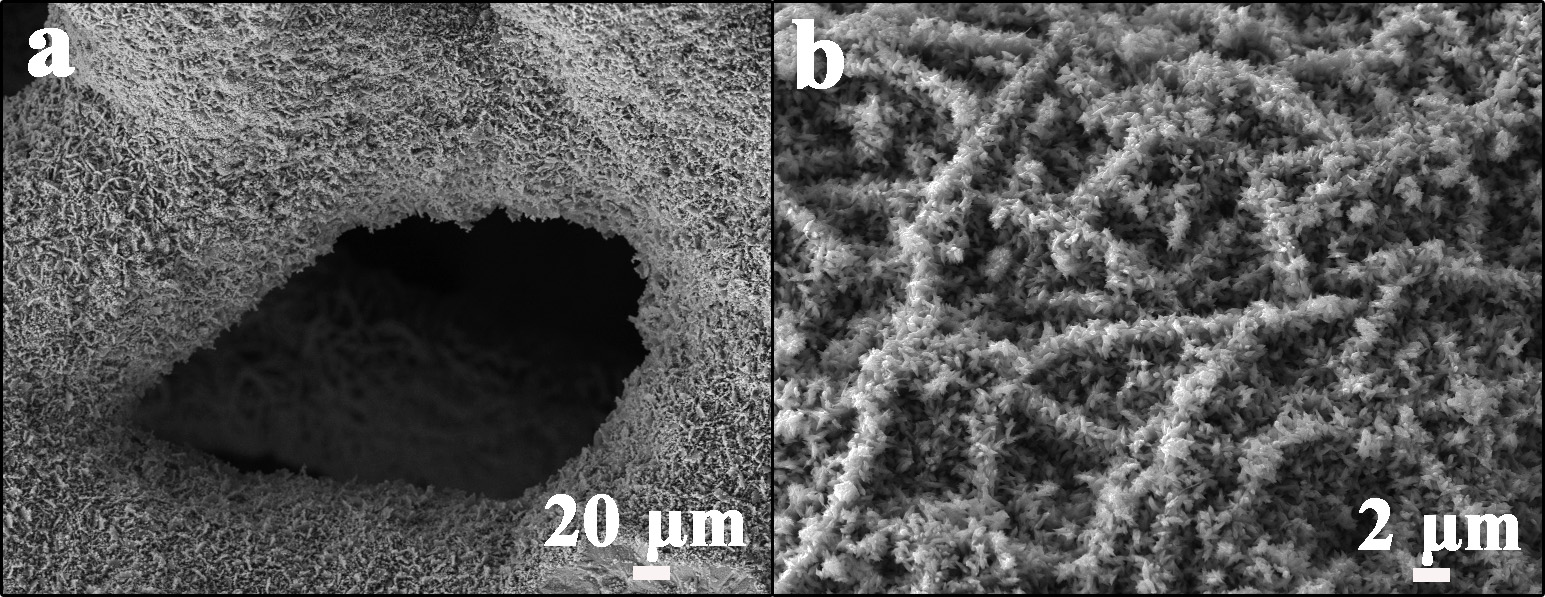
**

**Figure S5**. SEM images in low and high magnification of the mixed solution of MB@RB (c and d).

**
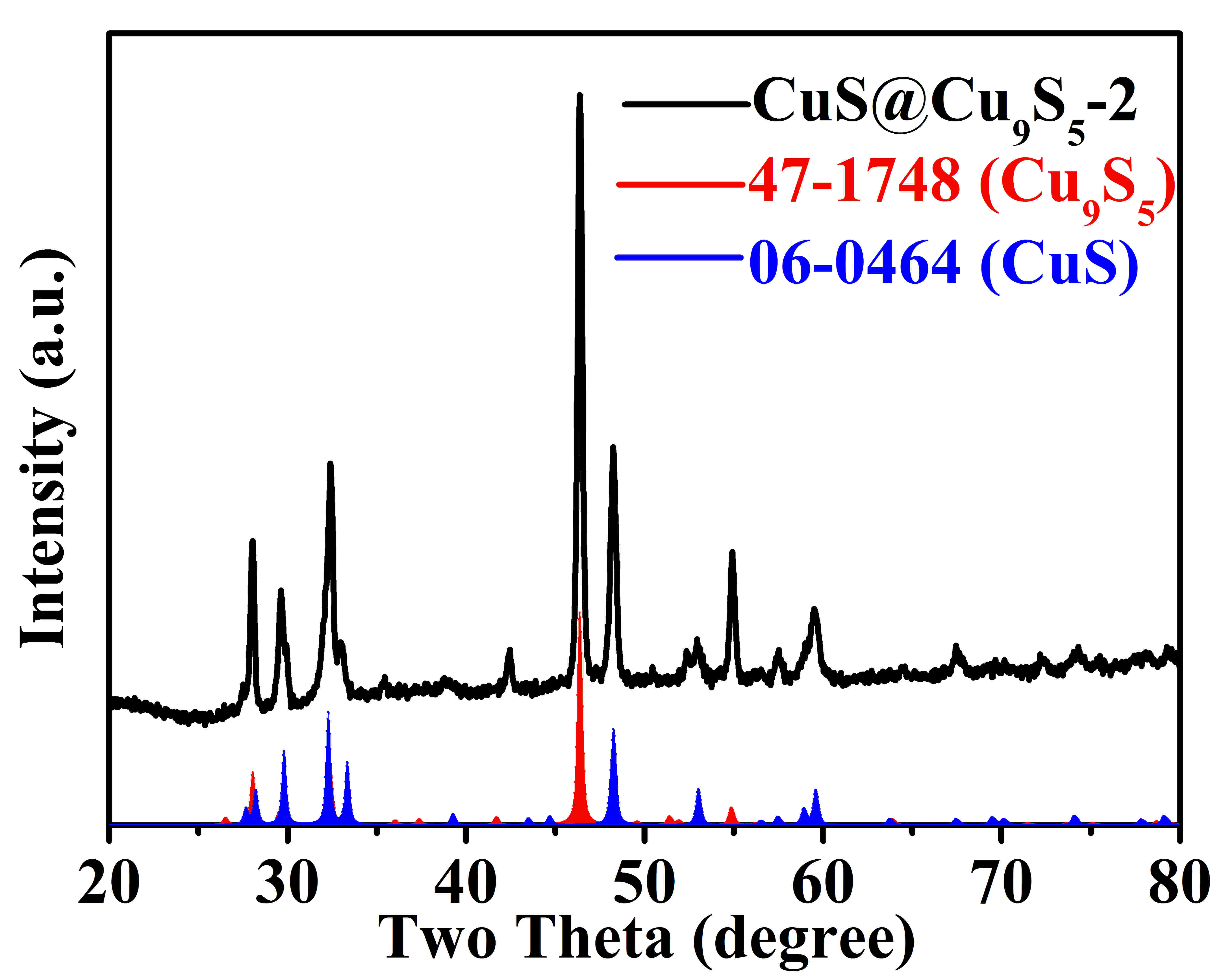
**

**Figure S6**. XRD pattern of CuS@Cu_9_S_5_-2 for the cation exchange using DMF as solvent.
